# Application of a physiologically based pharmacokinetic model to predict isoniazid disposition during pregnancy

**DOI:** 10.1111/cts.13614

**Published:** 2023-09-15

**Authors:** Ogochukwu U. Amaeze, Nina Isoherranen

**Affiliations:** ^1^ Department of Pharmaceutics University of Washington, School of Pharmacy Seattle Washington USA

## Abstract

Pregnancy can increase the risk of latent tuberculosis infection (LTBI) progression to tuberculosis (TB) disease. Isoniazid (INH) is the preferred preventative treatment for LTBI in pregnancy. INH is mainly cleared by N‐acetyltransferase 2 (NAT2) but the pharmacokinetics (PK) of INH in different NAT2 phenotypes during pregnancy is not well characterized. To address this knowledge gap, we used physiologically based pharmacokinetic (PBPK) modeling to evaluate NAT2 phenotype‐specific effects of pregnancy on INH disposition. A whole‐body PBPK model for INH was developed and verified for non‐pregnant NAT2 fast (FA), intermediate (IA), and slow (SA) acetylators. Model predictive performance was assessed using a drug‐specific model acceptance criterion for mean plasma area under the curve (AUC) and peak plasma concentration (*C*_max_), and the absolute average fold error (AAFE) for individual plasma concentrations. The verified model was extended to simulate INH disposition during pregnancy in NAT2 SA, IA, and FA populations. A sensitivity analysis was conducted using the verified PBPK model and known changes in INH disposition during pregnancy to determine whether NAT2 activity changes during pregnancy or other INH clearance pathways are altered. This analysis suggested that NAT2 activity is unchanged while other INH clearance pathways increase by ~80% during pregnancy. The model was applied to explore the effect of pregnancy on INH disposition in two ethnic populations with different NAT2 phenotype distributions and with high TB burden. Our PBPK model can be used to predict INH disposition during pregnancy in diverse populations and expanded to other drugs cleared by NAT2 during pregnancy.

**Study Highlights**

**WHAT IS THE CURRENT KNOWLEDGE ON THE TOPIC?**

Isoniazid (INH) is recommended for the treatment of latent tuberculosis infection in pregnant women. INH pharmacokinetics (PK) shows high inter‐individual variability arising from N‐acetyltransferase 2 (NAT2) polymorphism. Existing studies of the PK of INH in pregnancy are confounded by concurrent administration of other tuberculosis and antiretroviral medications and variable populations. The impact of pregnancy on the disposition of INH is poorly understood.

**WHAT QUESTION DID THIS STUDY ADDRESS?**

We employed a physiologically based pharmacokinetic (PBPK) modeling approach to investigate how the PK of INH changes during pregnancy in different NAT2 phenotypes and to delineate the processes that may contribute to changes in INH exposure during pregnancy. Additionally, our study demonstrates how pregnancy effects may vary in populations with different NAT2 phenotype distributions.

**WHAT DOES THIS STUDY ADD TO OUR KNOWLEDGE?**

The PBPK modeling suggests that the increased clearance of INH during pregnancy is due to a pregnancy‐mediated increase in renal clearance and non‐NAT2 activity (up to 80%). NAT2 activity appears unchanged during pregnancy; hence the increase in INH clearance due to pregnancy is greater in NAT2 poor metabolizers.

**HOW MIGHT THIS CHANGE CLINICAL PHARMACOLOGY OR TRANSLATIONAL SCIENCE?**

This work demonstrates that pregnancy does not significantly alter NAT2 activity. The results can be extrapolated to other drugs cleared by NAT2. The effect of NAT2 phenotype on INH disposition in pregnant and non‐pregnant individuals has implications for dosing regimen design in populations with high TB burden. Our PBPK model can be integrated with a pharmacodynamic model to optimize INH dosing regimens.

## INTRODUCTION

Tuberculosis (TB) infection and disease are caused by *Mycobacterium tuberculosis*. Infected persons are classified as having TB disease if clinical TB symptoms are present, or as having latent TB infection (LTBI) if their infection is asymptomatic. Latent TB has the possibility (10%) of reactivation and progression to TB disease. TB disease is an important non‐obstetric cause of mortality among women of reproductive age.^1^ During pregnancy, the estimated median prevalence of LTBI in low TB‐burden countries is 15%, while in high TB‐burden countries the estimated prevalence of LTBI is 30%–34%.^2^ Pregnancy‐induced suppression of immune response may impair responses to *M. tuberculosis*, thereby making the progression from LTBI to TB disease during pregnancy more likely.^3^, ^4^ Untreated or inadequately treated TB disease during pregnancy is associated with adverse pregnancy, maternal, and fetal outcomes, including miscarriage, pre‐eclampsia, premature birth, and congenital TB infection.^5^, ^6^ Thus, effective treatment of TB and prevention of the progression of LTBI to TB disease during pregnancy is critical for maternal and child health.

Isoniazid (INH) is a first‐line anti‐TB drug. Current guidelines for TB preventive therapy for pregnant women recommend 6–9 months of INH treatment.^7^ INH target serum peak concentration is 3–6 μg/mL after a 300 mg daily dose and 9–15 μg/mL after a 900 mg biweekly dose.^8^ A dose increase is recommended if the peak concentration is <2 μg/mL after the 300 mg dose or <7 μg/mL after the 900 mg dose.^9^ INH is absorbed rapidly after oral administration. It is minimally bound to plasma proteins (*f*_u_ = 0.95) and distributes to total body water with an apparent volume of distribution of 0.6 L/kg.^10^ INH is predominantly cleared through acetylation by the polymorphic N‐acetyltransferase 2 (NAT2; 50%–90%).^11^ Minor elimination pathways include renal elimination as unchanged drug, hydrolysis by amidases, and proposed oxidation by CYP2E1 and other unknown enzymes to reactive intermediates and oxoacids.^11^ Genetic polymorphisms in the NAT2 gene give rise to different acetylation phenotypes, and individuals are classified as NAT2 fast (FA), intermediate (IA), or slow (SA) acetylators.^12^ NAT2 genotype accounts for most of the inter‐individual variability in INH exposure.^13^ At present, minimal data are available on how NAT2 activity and expression are altered during pregnancy, preventing mechanistic predictions of INH disposition during pregnancy and making optimization of therapy during pregnancy challenging.

Pregnancy‐related changes in maternal physiology can alter drug pharmacokinetics (PK) and response. INH disposition during pregnancy is likely defined by both NAT2 status and pregnancy‐mediated changes in clearance processes. Several studies have explored the PK of INH during pregnancy.^14^, ^15^, ^16^ Following INH monotherapy (300 mg) to NAT2‐genotyped pregnant (*n* = 420) and postpartum (*n* = 637) women with HIV, population pharmacokinetics (popPK) analysis suggested a 26% increase in INH clearance during pregnancy compared to postpartum. A decrease in INH exposure antepartum was observed in all genotype groups.^14^ In contrast, when HIV‐positive, HIV‐treated pregnant women with TB (*n* = 29, *n* = 8 paired for pregnancy and postpartum) were treated with INH (4–6 mg/kg) in a four‐drug combination therapy, no effect of pregnancy on INH exposure was observed when all NAT2 genotypes were considered together.^15^ Similarly, the exposure to INH was similar in antepartum and postpartum women of mixed NAT2 phenotypes following treatment with 900 mg INH in combination with rifapentin.^16^ Taken together, these data show a need for better understanding of the impact of pregnancy on INH PK in the different NAT2 phenotypes and with different comedications.

Physiologically based pharmacokinetic (PBPK) modeling is increasingly used to characterize and predict the effects of physiological changes on drug disposition during pregnancy.^17^, ^18^, ^19^ We hypothesized that PBPK modeling can be used to define changes in INH PK during pregnancy. To test this hypothesis, a full PBPK model of INH was developed and verified in a non‐pregnant population with defined NAT2 phenotypes. The model was extrapolated to the pregnant population to assess the impact of NAT2 genotype and pregnancy on INH exposure and the impact of pregnancy on NAT2 activity. The developed model allows predictions of INH disposition during pregnancy in different populations and can be used to predict the disposition of other drugs cleared by NAT2 during pregnancy.

## METHODS

### Curation of INH PK studies and assessment of variability

All published human clinical PK studies available for INH were identified via a PubMed search (https://pubmed.ncbi.nlm.nih.gov/) carried out in August 2022. Search keywords were “isoniazid” AND “pharmacokinetics,” and only the ‘clinical trials’ article type was included in the search. A total of 151 articles were retrieved. As INH PK shows high inter‐individual variability resulting from NAT2 genetic polymorphisms, only clinical studies that reported NAT2 genotype or acetylation phenotype of the study participants and were conducted in healthy participants were considered for model development and verification. A total of 5 studies and 24 (5 intravenous [i.v.] and 19 oral [p.o.]) INH mean plasma concentration–time profiles were used for the model development and verification.^10^, ^20^, ^21^, ^22^, ^23^ (Table S1). Two i.v. and four p.o. dosing datasets,^21^ including NAT2 FA and NAT2 SA, were chosen as the training datasets for model development. Three i.v. and 15 p.o. dosing datasets in FA, IA, and SA populations were used as test datasets to verify the model. The published mean concentration–time data from these studies were obtained by digitizing the plasma concentration–time profiles using WebPlotDigitizer 4.3 (https://apps.automeris.io/wpd/).

### Model acceptance critera

Drug‐specific model acceptance criteria were calculated according to a previously reported method^24^, ^25^ considering intra‐study and inter‐study PK variability in human INH PK data using Equations (1), (2), (3):

(1) $$\sigma =\sqrt{\ln\left[{\left(\frac{CV\%}{100}\right)}^{2}+1\right]}$$

(2) $$A\bar{x}=\exp\left[\ln\left(\bar{x}\right)+4.26\frac{\sigma }{\sqrt{N}}\right]$$

(3) $$B\bar{x}=\exp\left[\ln\left(\bar{x}\right)-4.26\frac{\sigma }{\sqrt{N}}\right]$$

where CV% is the observed mean of the coefficient of variation (CV) of the area under the curve (AUC) or the peak plasma concentration (*C*_max_) values from all the PK studies considered; *σ* is the calculated variability of a given parameter; $\bar{x}$ is the observed mean AUC or *C*_max_ value; *N* is the mean number of participants in the clinical study considered; and A and B are the calculated absolute values for upper‐ and lower‐fold limits, respectively, for the given PK parameter.

The PK studies in NAT2 FA, IA, and SA summarized in Table S1 were used to calculate the upper‐ and lower fold limits for acceptable fold error. The calculated acceptance criterion ranges in the NAT2 FA, IA, and SA were 0.67–1.48‐fold, 0.85–1.17‐fold, and 0.50–1.97‐fold for AUC values, respectively, and 0.50–1.98‐fold, 0.61–1.63‐fold, and 0.28–3.56‐fold for *C*_max_ values, respectively. The simulated mean AUC and *C*_max_ values for each study were compared to the observed mean, and the acceptance range calculated around observed mean. The results were considered acceptable if the mean simulated values were within the acceptance range.

### Model development and parameterization in a non‐pregnant population

**FIGURE 1:**
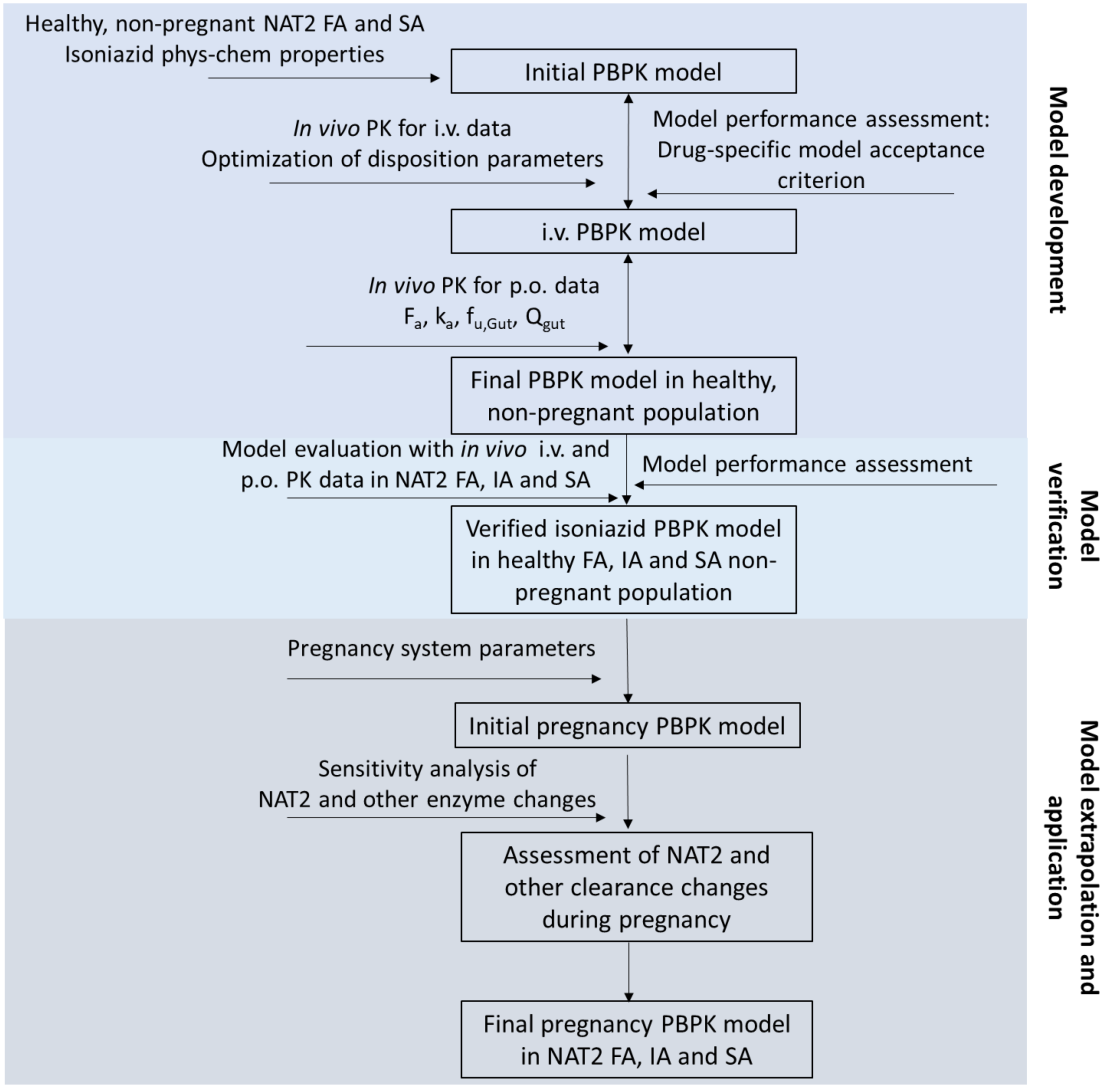
Workflow for the isoniazid physiologically based pharmacokinetic model development and verification in N‐acetyltransferase 2 fast, intermediate, and slow acetylators. Abbreviations: FA, fast acetylators; IA, intermediate acetylators; i.v., intravenous; NAT2, N‐acetyltransferase 2; PBPK, physiologically based pharmacokinetic; SA, slow acetylators.

A full PBPK model of INH was developed to simulate INH disposition in NAT2 FA, IA, and SA using a systematic model development and verification approach,^25^ and Simcyp simulator V21.0 (Certara UK Limited). The workflow for model development and verification is shown in Figure 1.

INH physicochemical input parameters were retrieved from a previously published INH PBPK model used to simulate INH PK in a non‐pregnant population.^26^ The values were verified independently before incorporation into the model. The systemic clearance values were first optimized for a non‐pregnant population using data collected from the literature and comparing the simulated and observed AUC values following i.v. dosing. The renal clearance (CL_r_) for INH is independent of NAT2 phenotype. The value for CL_r_ was calculated from the reported amount excreted unchanged to urine and the reported AUC, resulting in a value of 2.76 L/h.^27^ The overall hepatic CL (CL_hep_) was calculated from CL_hep_ = CL−CL_r_ using the observed systemic clearance of 25 and 10 L/h in FA and SA.^21^ This resulted in CL_hep_ of 22.24 L/h and 7.24 L/h in FA and SA, respectively. NAT2 SA is not a complete loss of function phenotype but has some remaining acetylation activity.^28^ The NAT2 activity in the common SA genotype group (NAT2*5) was calculated to be 12% of that in the FA genotype group based on in vitro data.^28^ The rest of the hepatic clearance (CL_other_) was assigned to a combination of enzymes including amidase, possible CYP2E1, and other oxidation enzymes.^11^ CL_other_ was assumed to be the same in FA and SA. The clearance value was calculated from the CL_hep_ determined above and the reported relative activity of NAT2 in SA and FA groups as shown in Equations (4), (5), (6):

(4) $${\mathrm{CL}}_{\mathrm{hep},\mathrm{FA}}={\mathrm{CL}}_{\text{other}}+{\mathrm{CL}}_{\mathrm{NAT}2,\mathrm{FA}}$$

(5) $${\mathrm{CL}}_{\mathrm{hep},\mathrm{SA}}={\mathrm{CL}}_{\text{other}}+0.12^{*}{\mathrm{CL}}_{\mathrm{NAT}2,\mathrm{FA}}$$

(6)

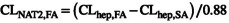

in which CL_NAT2,FA_ is the clearance due to NAT2 in FA, CL_hep,FA_ is the total hepatic clearance in FA, and CL_hep,SA_ is the total hepatic clearance in SA, assuming that INH is a relatively low extraction ratio (ER < 0.25) drug.

The unbound hepatic intrinsic clearance (CL_u,int,NAT2_) in the FA and SA was then back‐calculated from the hepatic CL_NAT2_ using the well‐stirred model of hepatic clearance. The CL_u,int,NAT2_ was calculated as 19.92 L/h and 2.39 L/h, in NAT2 FA and SA, respectively. The calculated CL_u,int,NAT2_ (L/h) was converted to CL_u,int,H,NAT2_ (μL/min/mg cytosolic protein) based on Equation 7:

(7) $$\begin{array}{c} {\mathrm{CL}}_{u,\mathrm{int},H,\mathrm{NAT}2}=\frac{{\mathrm{CL}}_{u,\mathrm{int},\mathrm{NAT}2}\;\times \;10^{6}}{\text{CPPGL}\;\times \;\text{Liver weight}\;\times \;60} \end{array}$$

in which CPPGL is cytosolic protein per gram of liver (Simcyp healthy population value 81.033 mg/g liver was used).

INH has been reported to show nonlinear kinetics at doses above 300 mg^22^, ^29^ and hence the Michaelis–Menten kinetic values for NAT2 were incorporated in the model to allow simulation of nonlinear kinetics if present. The reported *K*_m_ ^28^ value was used directly and the *V*_max_ was calculated from the CL_u,int,H,NAT2_ (μL/min/mg cytosolic protein) and reported *K*_m_. IA individuals have one fast (NAT2*4) and one slow (NAT2*5, *6 or *7) allele. *V*_max_ for this group was computed from 50% of CL_u,int,H,NAT2_ and *K*_m_ in FA (*4) and SA (*5). Separate models were generated for FA, IA, and SA individuals as the current version of Simcyp does not include NAT2 and its polymorphisms as a population variable. The calculated population‐specific *V*_max_ and *K*_m_ values were added in the cytosolic enzyme kinetics tab in the Simcyp simulator for the FA, IA, and SA models. The CL_other_ (L/h) was assigned as additional systemic clearance and was the same in the models.

Once the CL parameters were optimized, a tissue distribution model of INH was developed using a full‐body PBPK model, and the simulated plasma concentration–time curves were compared to the observed training datasets following i.v. dosing. Tissue‐to‐plasma partition coefficients (*K*_p_) were predicted according to the Rodger and Rowland method (method 2 in the Simcyp simulator). These values were used as such for the FA model. As the volume of distribution at steady state (*V*_ss_) has been reported to be different in SA and FA populations,^21^, ^30^, ^31^ the *K*_p_ values for two major distribution organs, adipose and skin, were optimized in the SA model to match clinically observed *V*_ss_ values and to recover the concentration–time profile after i.v. administration in SA.^21^

Once the simulated plasma concentration–time curves met the predefined model acceptance criterion after i.v. administration, the oral absorption parameters (*F*_a_, *k*_a_, *f*_uGut_, *P*_eff,man,_ and *Q*_Gut_) were incorporated into the model. A first‐order absorption model was used to describe INH oral absorption, and the absorption input parameters were used as reported previously.^26^ The final model input parameters are shown in Table S2.

The model quality was assessed via the model acceptance criterion for AUC and *C*_max,_ and the accuracy of distribution kinetics via the absolute average fold error (AAFE) calculated from individual concentration points as:

$$\text{AAFE}=10^{\frac{1}{n}\sum \left|\log\left(\frac{\text{predicted}}{\text{observed}}\right)\right|}$$

An AAFE ≤ 1.5 criterion was chosen as successful prediction of distribution kinetics.

### Model verification in a healthy, non‐pregnant population

The model was verified by simulating INH plasma concentration–time profiles after i.v. and p.o. dosing and comparing the simulated data to observed data from clinical studies (test datasets) which were not used in the model development. All simulations were carried out using the healthy, non‐pregnant virtual population within the Simcyp Simulator version 21. For each simulation, the characteristics of the virtual population were set to match the observed data in terms of reported sex, age range, number of participants, route of administration, and dose. A male:female ratio of 1:1 and age range of 20–55 years were used for studies that did not provide demographic information. Ten trials were simulated for each study to ensure the representation of the clinical study participants in the virtual population^32^ and to capture inter‐study variability. The model performance was assessed using the model acceptance criterion and AAFE as described above.

### Pregnancy PBPK model, sensitivity analysis, and simulations

Once the INH PBPK model was verified in the non‐pregnant population, the Simcyp library pregnancy population model was used to explore the impact of pregnancy on INH exposure. With no knowledge of pregnancy‐mediated changes in NAT2, and other INH‐clearing enzyme activity, the model was first used with no changes in any of the INH metabolic clearance pathways to assess whether known pregnancy‐related physiological changes alone could explain INH disposition during pregnancy. A sensitivity analysis of INH disposition was then conducted to investigate how specific enzyme activity during pregnancy impacts INH disposition in the different NAT2 phenotypes. This was done by varying NAT2 *V*_max_ and CL_other_ during pregnancy in the range of 110%–200% of non‐pregnant values and assessing the impact of the altered values on INH oral clearance. INH clearance reported during pregnancy in a clinical study in NAT2 SA, IA, and FA^14^ was used as a reference for INH clearance during pregnancy to delineate the impact of pregnancy on NAT2 and other enzyme activity in different NAT2 phenotypes.

The model incorporating the change in CL_other_ during pregnancy was applied to evaluate whether INH *C*_max_ in pregnant NAT2 FA, IA, and SA is within the recommended therapeutic range of 3–6 mg/L after a 300 mg daily dose. The model was also used to evaluate variability in pregnancy effects on INH disposition in different geographical regions with different ethnicities. Hypothetical pregnant populations were built for Africa (AFR) and South Asia (SAS) regions using the reported NAT2 phenotype distributions.^28^ The AFR and SAS regions were selected due to their high TB burden. The NAT2 phenotype frequencies implemented in the pregnant populations were FA: 0.2, IA: 0.48, and SA: 0.32 for the AFR region, and FA: 0.059, IA: 0.331, and SA: 0.609 for the SAS region. For all the simulations, a trial design of 100 participants (10 × 10 trials) aged 18–45 years, administered a single oral 300 mg dose of INH in the fasted state was used.

## RESULTS

### Isoniazid PBPK model development and verification

**FIGURE 2:**
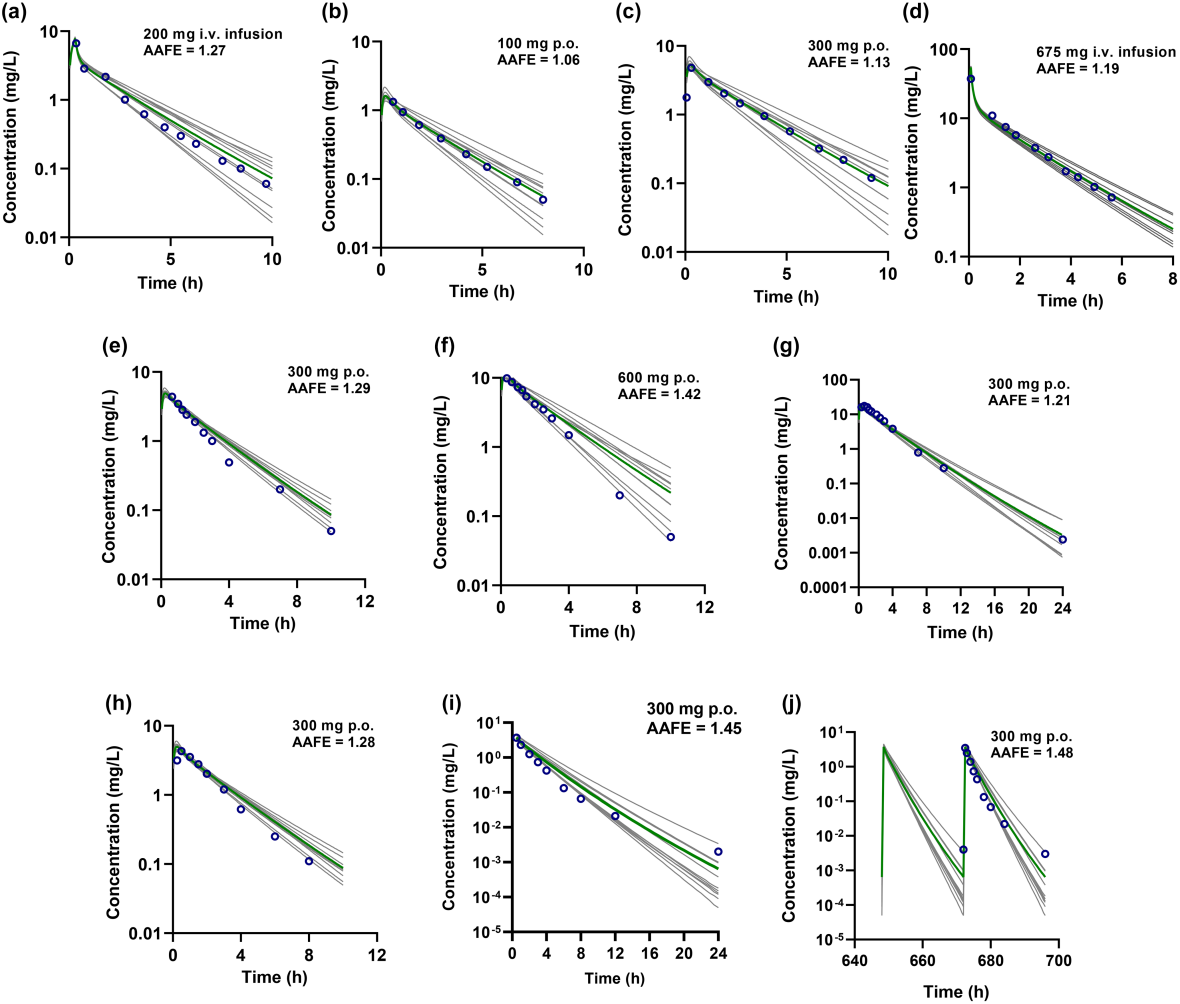
Simulated plasma concentration–time profiles following intravenous (i.v.) and oral (p.o.) administration of isoniazid (INH) overlaid with mean observed plasma INH concentrations in non‐pregnant N‐acetyltransferase 2 (NAT2) fast acetylators (FA). Open circles represent the mean observed data, gray solid lines show simulated plasma concentrations in 10 trials, and the green solid line represents the mean of the trials. The mean observed INH concentrations are from Kinzig‐Schippers et al.^21^ (a–c); Boxenbaum and Riegelman^10^ (d); Kubota et al.^22^ (e–g); Bing et al.^20^ (h); and Yoo et al.^23^ (i‐j). (a–c) were used for model development and (d–j) were used for model verification. The insets show the dose and route of administration in each study and the absolute average fold error (AAFE) calculated from the mean simulated and observed concentrations of each respective dataset.

**FIGURE 3:**
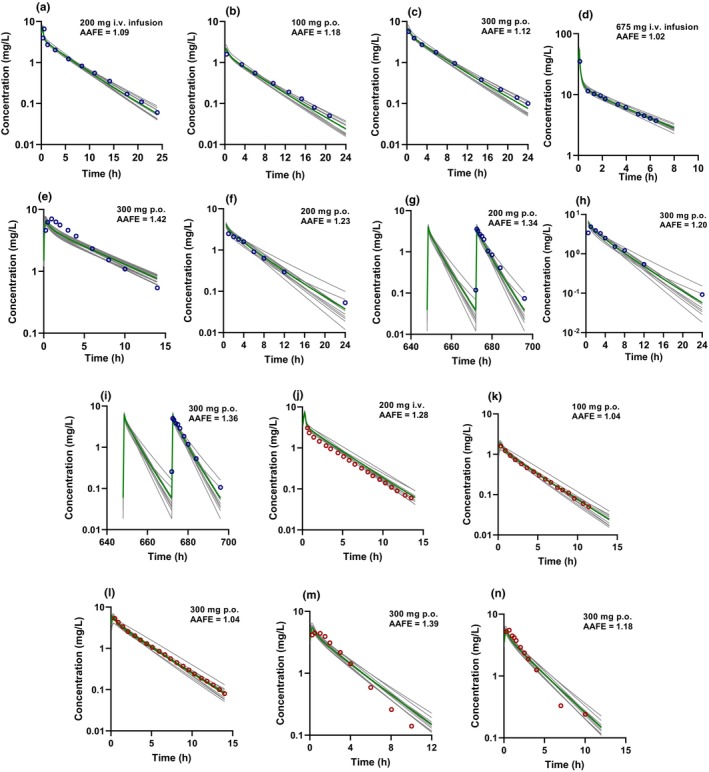
Simulated plasma concentration–time profiles following intravenous (i.v.) and oral (p.o.) administration of isoniazid (INH) overlaid with mean observed plasma INH concentrations in non‐pregnant N‐acetyltransferase 2 (NAT2) intermediate acetylators (IA) and slow acetylators (SA). Open circles (purple: SA, red: IA) represent the mean observed data, gray solid lines show simulated plasma concentrations in 10 trials, and the green solid line represents the mean of the trials. The mean observed INH concentrations are from Kinzig‐Schippers et al.^21^ (a–c, j–l); Boxenbaum and Riegelman^10^ (d); Bing et al.^20^ (e, m); and Yoo et al.^23^ (f–i). (a–c) were used for model development and (d–n) were used for model verification. The insets show the dose and route of administration in each study and the absolute average fold error (AAFE) calculated from the simulated and mean observed concentrations of each respective dataset.

The INH PBPK model was first developed and optimized with a training dataset comprised of i.v. and p.o. dosing in NAT2 FA, IA, and SA (Table S1). The model recovered the concentration–time profiles of the training set studies and captured the *C*_max_ and *t*_max_ well (Figures 2 and 3). All the simulated AUC and *C*_max_ values of the training set met the predefined INH‐specific model acceptance criteria (Table 1). The AAFE values were 1.07–1.27 and within the acceptance criterion with good similarity between the simulated and observed plasma concentration–time profiles (Figures 2 and 3). The models were verified using independent test datasets for NAT2 FA, IA, and SA, including single i.v. and p.o. dosing and multiple p.o. dosing regimens. Representative model input and output files for FA, IA and SA populations are included in Supplemental Datafiles S1‐S3. Like the training dataset, the model adequately recovered the observed data from the test dataset. All the simulated AUC and *C*_max_ values were within the model acceptance criteria for those studies, and the AAFE values for the plasma concentration–time data were within the predefined acceptance criteria. Overall, the model performance showed successful development and verification for non‐pregnant adults who are NAT2 FA, IA, and SA.

**TABLE 1:** Predicted and observed isoniazid (INH) area under the curve (AUC) and peak plasma concentration (*C*_max_) values for the studies used for INH physiologically based pharmacokinetic (PBPK) model development (training set) and verification (test dataset).

Dose	Phenotype	AUC (mg.h/L)			*C*_max_ (mg/L)			Reference
		Mean observed	Acceptance range	Mean predicted	Mean observed	Acceptance range	Mean predicted	
**Training dataset**								
iv. inf, 20 min, 200 mg	FA	8.0	5.36–11.84	10.60	5.2	2.60–10.30	7.83	21
po, sd, 100 mg	FA	3.1	2.08–4.59	3.42	1.2	0.60–2.38	1.64	21
po, sd, 300 mg	FA	12.3	8.24–18.20	11.89	5.4	2.70–10.69	5.39	21
iv. inf, 20 min, 200 mg	SA	20.4	10.20–40.19	21.30	6.8	1.90–24.21	8.42	21
po, sd, 100 mg	SA	9.1	4.55–17.93	8.96	2.1	0.59–7.48	2.20	21
po, sd, 300 mg	SA	32.1	16.05–63.24	27.86	7.8	2.18–27.77	6.74	21
**Test dataset**								
iv. inf, 20 min, 200 mg	IA	10.6	9.01–12.40	13.25	6.0	3.66–9.78	7.75	21
po, sd, 100 mg	IA	4.8	4.08–5.62	5.09	1.3	0.79–2.12	1.85	21
po, sd, 300 mg	IA	17.1	14.54–20.01	16.50	6.15	3.75–10.02	5.85	21
po, sd, 300 mg	IA	14.24	12.10–16.66	15.82	6.77	4.13–11.04	5.45	22
po, sd, 300 mg	IA	16.34	13.89–19.12	15.83	5.72	3.49–9.32	5.48	20
iv. inf, 5.2 min 670 mg	FA	35.65	23.89–52.76	38.42	37.16	18.58–73.58	56.81	10
po, sd, 300 mg	FA	9.54	6.39–14.12	11.65	5.72	2.86–11.33	4.99	22
po, sd, 600 mg	FA	24.99	16.74–36.99	26.70	13.92	6.96–27.56	11.72	22
po, sd, 900 mg	FA	48.19	32.29–71.23	45.24	21.49	10.75–42.55	17.93	22
po, sd, 300 mg	FA	10.35	6.93–15.32	11.66	4.93	2.47–9.76	5.06	20
po, sd, 300 mg	FA	6.29	4.21–9.31	9.82	3.74	1.87–7.41	4.28	23
po, md, 300 mg	FA	6.66	4.46–9.86	10.01	3.58	1.79–7.09	4.43	23
iv. inf, 5.4 min, 670 mg	SA	55.46	27.73–109.26	59.61	35.08	9.82–124.88	55.65	10
po, sd, 300 mg	SA	42.24	21.12–83.21	32.68	8.28	2.32–29.48	6.57	20
po, sd, 200 mg	SA	15.10	7.55–29.75	18.05	3.05	0.85–10.86	4.88	23
po, md, 200 mg	SA	18.90	9.45–37.23	18.31	4.06	1.14–14.45	4.92	23
po, sd, 300 mg	SA	27.0	13.50–53.19	27.52	5.0	1.40–17.80	7.38	23
po, md, 300 mg	SA	28.0	14.00–55.16	27.92	6.57	1.84–23.39	7.44	23

*Note*: The observed AUC and *C*_max_ for Reference 10 were obtained from noncompartmental analysis of the digitized observed data.

Abbreviations: AUC, area under the curve; *C*_max_, peak plasma concentration; FA, fast acetylators; IA, intermediate acetylators; inf, infusion; i.v., intravenous; md, multiple doses; p.o., oral; SA, slow acetylators; sd, single dose.

### Simulation of isoniazid PK in a pregnant population

The verified model in the non‐pregnant population was applied to evaluate the impact of pregnancy on NAT2 and other enzyme activity, and to predict INH disposition during pregnancy. The observed increase in INH clearance in pregnant women has been reported as 16%, 21%, and 30% in NAT2 FA, IA, and SA, respectively.^14^ When INH disposition during pregnancy was simulated, assuming no change in NAT2 or other INH clearing enzyme activity during pregnancy, the changes in INH CL were underpredicted in all three populations (5%, 7.3%, and 11% increase predicted in FA, IA, and SA, respectively). We hypothesized that the additional observed increase in INH clearance during pregnancy could be attributed to either increased NAT2 activity or CL_other_ during pregnancy. This hypothesis was tested via a sensitivity analysis that was carried out by varying NAT2 activity and CL_other_ to recover the observed INH clearance change in NAT2 FA, IA, and SA populations. The sensitivity analysis suggests that the CL_other_ increases by ~80% while NAT2 activity is unchanged at the second trimester of pregnancy (Table 2). When this change in CL_other_ was incorporated into the model and INH plasma concentrations in the pregnancy population simulated, the refined model predicted clearance changes comparable to the observed data (Table S3). This suggests that NAT2 activity is unchanged during pregnancy while non‐NAT2 activity increases.

**TABLE 2:** Sensitivity analysis of the changes in NAT2 and other INH clearing enzyme activity during pregnancy.

Increase (%)	Predicted CL (L/h) in FA	% change^a^	Predicted CL (L/h) in SA	% change^a^
Other enzyme clearance				
0	33.9	0	12.3	0
10	36.3	6.4	14.3	14.0
20	36.8	7.6	14.7	16.8
30	37.3	8.9	15.2	19.4
40	37.8	10.1	15.7	21.9
50	38.8	12.5	16.2	24.2
60	39.3	13.6	16.6	26.4
70	39.7	14.4	17.1	28.4
80	40.2	15.5	17.6	30.4
NAT2 *V*_max_				
0	33.9	0	12.3	0
10	39.2	13.2	13.5	9.1
20	42.3	19.7	13.9	11.7
30	45.1	24.7	14.3	14.1
40	48.4	29.7	14.7	16.4
50	51.7	34.2	15.1	18.6
60	55.0	38.3	15.5	20.7
70	58.5	41.9	15.9	22.7
80	62.0	45.2	16.3	24.7

*Note*: Predicted isoniazid clearances in fast acetylators and slow acetylators with varying NAT2 and other enzyme clearance values are shown.

^a^ Percent change in clearance during pregnancy (GW28) compared to healthy non‐pregnant female.

**FIGURE 4:**
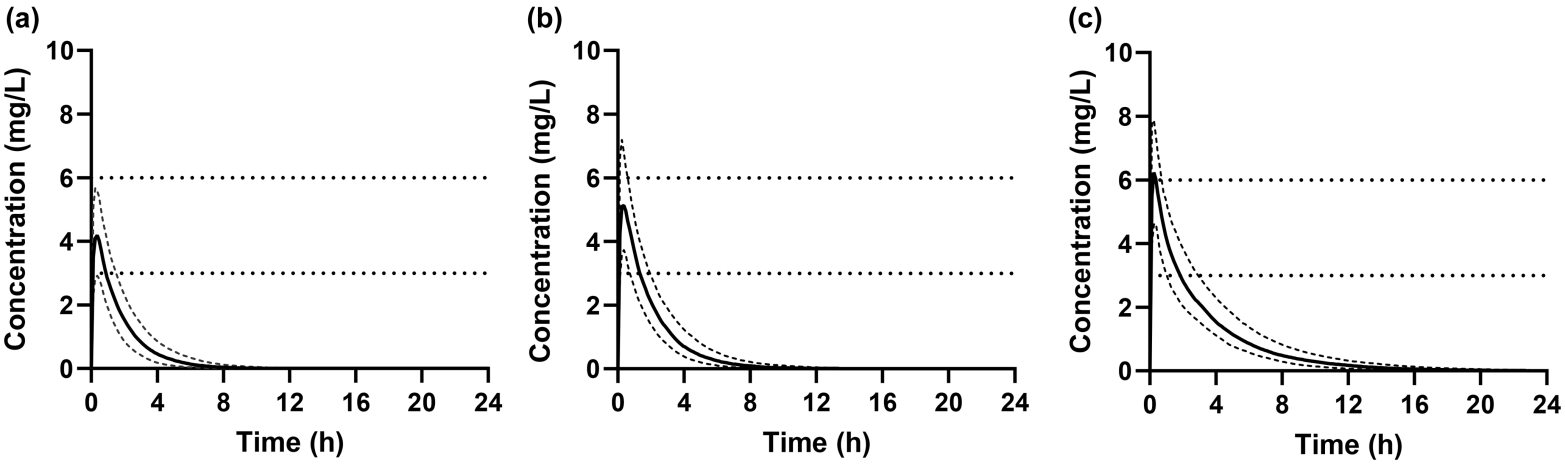
Simulated plasma concentration–time profiles of isoniazid (INH) in N‐acetyltransferase 2 (NAT2) (a) fast acetylators, (b) intermediate acetylators, and (c) slow acetylators during pregnancy (gestational week 28) following administration of a single 300 mg INH dose. The solid line shows the mean plasma concentration of the simulated population, and the dashed lines show the 10th and 90th percentiles of the simulated data. The dotted horizontal lines represent the recommended therapeutic C_max_ range for isoniazid following a 300 mg oral dose.

The model was applied to test whether INH *C*_max_ in the different NAT2 phenotypes during pregnancy is expected to be within the concentration range (3–6 mg/L) recommended for effective TB therapy. The predicted mean *C*_max_ in the NAT2 FA was 4.4 ± 1.1 mg/L with 12% of the population below the recommended effective *C*_max_, and 7% above the *C*_max_ threshold. In the NAT2 IA, the predicted mean *C*_max_ was 5.3 ± 1.2 mg/L, 28% of the population had *C*_max_ values above 6 mg/L, and 1% was below 3 mg/L. The predicted mean *C*_max_ in the NAT2 SA was 6.3 ± 1.3 mg/L. None of the SA population was below the recommended effective *C*_max_ but 56% of the population had a *C*_max_ above 6 mg/L (Figure 4).

**FIGURE 5:**
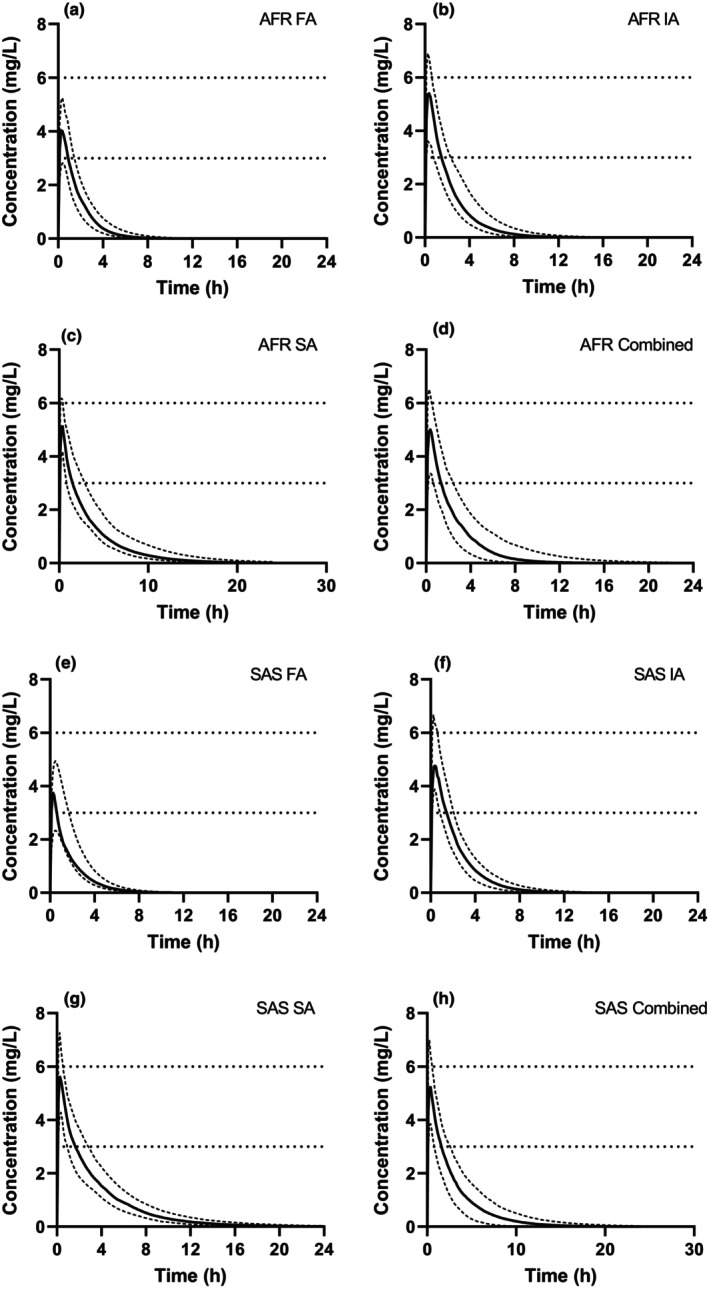
Simulated plasma concentration–time profiles of isoniazid (INH) following oral (p.o.) administration of a single 300 mg INH dose in pregnant (gestational week 28) populations with various N‐acetyltransferase 2 (NAT2) phenotype distributions. The populations shown are (a) Africa (AFR) fast acetylators (FA), (b) Africa intermediate acetylators (IA), (c) Africa slow acetylators (SA), (d) Africa combined NAT2 phenotypes, (e) South Asia (SAS) FA, (f) South Asia IA, (g) South Asia SA, and (h) South Asia combined NAT2 phenotypes. The solid lines show the population mean simulated plasma concentration, and the dashed lines show the 10th and 90th percentiles of the simulated data. The horizontal dotted lines represent the recommended therapeutic range (3–6 mg/L) for isoniazid C_max_ following a 300 mg oral dose.

To evaluate how pregnancy effect would vary in populations with different NAT2 phenotype distributions, INH disposition was simulated in a pregnant population reflecting NAT2 phenotype distribution in two high TB‐burden regions. In the AFR population having 20% FA, 48% IA, and 32% SA, 26% of the population had a *C*_max_ above the 6 mg/L while 5% of the population had a *C*_max_ below 3 mg/L. In contrast, for the SAS population with 6% FA, 33% IA, and 61% SA, 41% of the population had a *C*_max_ above 6 mg/L and 2% had a *C*_max_ below 3 mg/L (Figure 5).

## DISCUSSION

Mechanistic PBPK modeling has been leveraged to support optimal dosing regimen design in various special populations.^33^ We employed a PBPK modeling approach to assess the impact of pregnancy on INH PK in NAT2 FA, IA, and SA and to identify the drivers of INH PK changes during pregnancy. The PBPK model predicted INH PK parameters successfully in the non‐pregnant population across a wide dose range and in different NAT2 phenotypes, indicating that all the major processes including the nonlinear kinetics driving INH PK were adequately described in the model.

Little is known about the gestational age‐dependent activity of NAT2, amidases, and CYP2E1. The Simcyp pregnancy population includes gestational age‐dependent changes in physiology, CL_r_, and activity of major enzymes such as CYP1A2, CYP2D6, and CYP3A4.^34^ The initial PBPK model, accounting for only these pregnancy‐mediated physiological changes, predicted a 5%, 7.3%, and 11% increase in INH clearance during pregnancy in the FA, IA, and SA, respectively, likely due to increased glomerular filtration rate and CL_r_ of INH, but underpredicted the observed increase in INH clearance (16%, 21%, and 30% in the FA, IA, and SA, respectively).^14^ This suggests that pregnancy‐related physiologic changes alone do not explain the observed increased INH clearance, and other elimination mechanisms contribute to the increased INH clearance during pregnancy.

To determine the other contributing factors to INH clearance changes in pregnancy, a sensitivity analysis of the effect of changing CL_NAT2_ and CL_other_ on INH oral clearance was performed. The sensitivity analysis suggested that the observed difference in oral clearance is likely due to an increased CL_other_ with little or no change in NAT2 activity during pregnancy. To recover the observed change in INH clearance during pregnancy in FA and SA populations, CL_other_ has to increase by ~80% during pregnancy. It is likely that most of the CL_other_ is by amidases. Amidases are hydrolases localized in the blood and liver that metabolize drugs such as INH, pyrazinamide, and metopimazine.^35^, ^36^ Even though the role of amidases in INH elimination is well documented, the specific amidases and their relative contribution of INH clearance is not well defined.^37^ It is possible that multiple amidases in the liver or in other organs, or other enzymes such as CYP2E1 are also induced during pregnancy, potentially contributing to the increased INH clearance.

A higher level of amidase activity or SA status has been proposed to lead to a higher incidence of hepatotoxicity after INH dosing due to reduced acetylation and greater formation of reactive metabolites, including hydrazine.^38^ Whether the apparent increased activity of amidases during pregnancy increases the likelihood of hepatotoxicity remains to be established. An earlier study of INH preventive therapy in HIV‐positive women suggested greater adverse pregnancy outcomes when treatment was initiated during pregnancy than postpartum,^39^ a finding potentially explained by induction of amidases during pregnancy. Further studies to assess exposure to toxic INH metabolites in pregnant women who are NAT2 IA or SA are warranted.

Our observation of no change in NAT2 activity during pregnancy is congruent with an earlier report of NAT2 activity in pregnant women using caffeine urinary metabolic ratios.^40^ This study showed that mean NAT2 activity was unchanged during mid and late pregnancy. Collectively, these findings suggest that pregnancy does not increase the activity of NAT2.

A paramount concern of drug therapy during pregnancy is the attainment of optimal drug concentrations that maximize therapeutic benefit while minimizing toxicity or adverse effects. Low concentrations of INH correlate with poorer clinical and treatment outcomes.^9^ Understanding the therapeutic consequences of the observed reduced INH exposure during pregnancy is essential, especially in the face of high inter‐individual variability in INH PK. INH peak concentration of 3–6 μg/mL after 300 mg daily doses is generally accepted as the concentration required for effective therapy and is widely used for INH therapeutic drug monitoring.^8^ However, lower INH peak plasma concentrations have been reported in different geographical populations with different NAT2 genotype frequency distributions.^41^, ^42^ Similarly, variable INH concentrations and exposure were reported in various pregnant populations.^14^, ^15^, ^16^, ^43^ Invariably, plasma concentrations required for effective TB therapy during pregnancy are not well defined. It is possible that due to altered immune response during pregnancy the target concentrations are also different for most effective therapy. Our assessment of INH PK during pregnancy in FA and SA showed a higher proportion (44%) of SA with *C*_max_ above the therapeutic range compared to FA (7%). Conversely, 12% of the FA had a *C*_max_ below the therapeutic range. This observation alludes to the growing recommendation for genotype‐guided dosing for INH^23^ and other NAT2 substrates.^44^ The evaluation of pregnancy effect on INH disposition showed high inter‐individual variability in the two populations with different NAT2 phenotype distributions and supports the need to define optimal INH concentrations and guide TB preventive therapy in pregnant individuals.

Our study provides an excellent example of applying PBPK modeling to assess drug disposition during pregnancy. However, our study has some limitations. We used a healthy pregnancy population and not a TB or TB‐HIV disease population, but our model could not be verified against data from clinical studies in healthy pregnant populations as such data are lacking. Drug disposition may be different between healthy and TB or HIV disease populations. A recent systemic review showed that INH dose‐normalized *C*_max_ and AUC estimates were higher in healthy volunteers than in patients with TB.^45^ Persons with HIV infection have an increased risk of progressing from LTBI to TB disease and hence require INH preventive therapy. The concurrent use of antiretroviral treatment can impact INH disposition due to drug–drug interactions. The data of INH PK in pregnant NAT2‐genotyped women used here is predominantly (88%) from patients also treated with efavirenz.^14^ Efavirenz coadministration decreased INH AUC by 29% in NAT2 FA but had no effect on INH AUC in SA.^46^ In another study in which participants were not NAT2‐genotyped, efavirenz coadministration also decreased INH exposure by 29%.^47^ The absolute predicted INH clearance in FA and IA populations during pregnancy and postpartum based on the PBPK model was lower than that observed, likely due to the inducing effect of efavirenz on INH clearance.

In conclusion, we developed and verified a PBPK model for INH in healthy FA, IA, and SA individuals, which was extrapolated to the pregnancy population. Our PBPK model could explain the possible factors contributing to the observed increased INH clearance in pregnancy and was applied to evaluate INH PK during pregnancy in populations with varying NAT2 phenotype distributions. The developed pregnancy model can be applied to quantitatively predict the disposition of other NAT2 substrates such as hydralazine used during pregnancy. Future expansion of the model could incorporate TB‐HIV disease, drug–drug interactions, and pregnant populations to explore the interplay of all these factors on the disposition of INH.

## AUTHOR CONTRIBUTIONS

O.U.A. and N.I. wrote the manuscript. O.U.A. and N.I. designed the research. O.U.A. performed the research. O.U.A and N.I. analyzed the data.

## FUNDING INFORMATION

This study was supported by the Bill & Melinda Gates Foundation (grant INV‐029091).

## CONFLICT OF INTEREST STATEMENT

N.I. reports consultancy agreements with Boehringer‐Ingelheim and Johnson and Johnson, and honoraria from ASPET and McGraw Hill. O.U.A. has declared no competing interests for this work.

## Supporting information


Data S1

Data S2

Data S3

Tables S1‐S3
