# Supplementary material for: Application of a physiologically based pharmacokinetic model to predict isoniazid disposition during pregnancy
Source: Clin Transl Sci. 2023 Sep 15;16(11):2163–76. doi: 10.1111/cts.13614 (PMC10651660; doi:10.1111/cts.13614)
Supplement: Supplementary file 4 — Tables S1‐S3 [file CTS-16-2163-s003.docx]

**Supplementary Material for:**

Application of a physiologically based pharmacokinetic model to predict isoniazid disposition during pregnancy.

**Authors:**

Ogochukwu U Amaeze, Nina Isoherranen*

**Authors’ Affiliation:**

Department of Pharmaceutics, University of Washington, School of Pharmacy, Seattle, Washington, United States.

**^*^Corresponding Author Information:**

Nina Isoherranen, PhD, Department of Pharmaceutics, University of Washington, Health Science Building Room H272M, Box 357610, Seattle WA 98195-7610, Phone: 206-543-2517, Fax: 206-543-3204, email: ni2@uw.edu.

**Supplemental tables**

**Table S1: Clinical studies and datasets used for isoniazid model development and verification in NAT2 fast (FA), intermediate (IA) and slow (SA) acetylators.**

| **Study** | **Number of participants** | **NAT2 phenotype** | **Dose**  **(mg)** | **Route of administration** | **Age (years)** | **Weight (kg)** |
| --- | --- | --- | --- | --- | --- | --- |
| **Training data set** | |  |  |  |  |  |
| Kinzig-Schippers et al., 2005 | 3 | FA | 200 | iv infusion, 20 min | 24 – 36 | 64 – 84 |
| Kinzig-Schippers et al., 2005 | 3 | FA | 100 | po, sd, | 24 – 36 | 64 – 84 |
| Kinzig-Schippers et al., 2005 | 3 | FA | 300 | po, sd, | 24 – 36 | 64 – 84 |
| Kinzig-Schippers et al., 2005 | 13 | SA | 200 | iv infusion, 20 min | 24 – 36 | 64 – 84 |
| Kinzig-Schippers et al., 2005 | 13 | SA | 100 | po, sd, | 24 – 36 | 64 – 84 |
| Kinzig-Schippers et al., 2005 | 13 | SA | 300 | po, sd, | 24 – 36 | 64 – 84 |
| **Test data set** | |  |  |  |  |  |
| Kinzig-Schippers et al., 2005 | 2 | IA | 200 | iv infusion, 20 min | 24 – 36 | 64 – 84 |
| Kinzig-Schippers et al., 2005 | 2 | IA | 100 | po, sd, | 24 – 36 | 64 – 84 |
| Kinzig-Schippers et al., 2005 | 2 | IA | 300 | po, sd, | 24 – 36 | 64 – 84 |
| Kubota et al., 2007 | 8 | IA | 300 | po, sd, | 21 – 25 | 56 – 71 |
| Bing et al., 2011 | 8 | IA | 300 | po, sd, | 22 – 26 | 59 – 69 |
| Boxenbaum and Riegelman, 1974^$^ | 1 | FA | 675 | iv infusion, 5.2 min | NR | NR |
| Kubota et al., 2007 | 8 | FA | 300 | po, sd, | 21 – 25 | 56 – 71 |
| Kubota et al., 2007 | 8 | FA | 600 | po, sd, | 21 – 25 | 56 – 71 |
| Kubota et al., 2007 | 8 | FA | 900 | po, sd, | 21 – 25 | 56 – 71 |
| Bing et al., 2011 | 8 | FA | 300 | po, sd, | 22 – 26 | 59 – 69 |
| Yoo et al., 2021 | 8 | FA | 300 | po, sd, | 20 – 45 | NR |
| Yoo et al., 2021 | 8 | FA | 300 | po, md | 20 – 45 | NR |
| Boxenbaum and Riegelman, 1974 | 1 | SA | 675 | iv infusion, 5.4 min | NR | NR |
| Bing et al., 2011 | 24 | SA | 300 | po, sd, | 22 – 26 | 59 – 69 |
| Yoo et al., 2021 | 3 | SA | 200 | po, sd, | 20 – 45 | NR |
| Yoo et al., 2021 | 3 | SA | 200 | po, md, | 20 – 45 | NR |
| Yoo et al., 2021 | 5 | SA | 300 | po, sd, | 20 – 45 | NR |
| Yoo et al., 2021 | 5 | SA | 300 | po, md, | 20 – 45 | NR |

^$^Study participants were not genotyped for NAT2 alleles, but phenotyped for acetylator status.

The participants in the other studies were genotyped for NAT2 Alleles. iv, intravenous; po, oral; sd, single dose; md, multiple dose

**Table S2: Isoniazid PBPK model input parameters**

| **Parameter** | **Value** | | |
| --- | --- | --- | --- |
| **Physicochemical** |  | | |
| Molecular weight (g/mol) | 137.14^a^ | | |
| LogP | -0.7^a^ | | |
| Compound type | Monoprotic base | | |
| pK_a_ | 1.82^a^ | | |
| B/P | 0.825^a^ | | |
| f_u,p_ | 0.95^a^ | | |
| **Absorption** |  | | |
| Model | First-order | | |
| F_a_ | 1^a^ | | |
| k_a_ (h^-1^) | 3.55^a^ | | |
| f_u,gut_ | 1^a^ | | |
| Q_Gut_ (L/h) | 2.516^a^ | | |
| P_eff,,man_ (10^-4^ cm/s) | 0.318^a^ | | |
| **Distribution** |  | | |
| Model | Full PBPK | | |
| K_p_ | **FA** | **IA** | **SA^b^** |
| Adipose | 0.17 | 0.17 | 0.65 |
| Bone | 0.43 | 0.43 | 0.43 |
| Brain | 0.78 | 0.78 | 0.78 |
| Gut | 0.71 | 0.71 | 0.71 |
| Pancreas | 0.76 | 0.76 | 0.76 |
| Heart | 0.74 | 0.74 | 0.74 |
| Kidney | 0.76 | 0.76 | 0.76 |
| Liver | 0.74 | 0.74 | 0.74 |
| Lung | 0.78 | 0.78 | 0.78 |
| Muscle | 0.73 | 0.73 | 0.73 |
| Skin | 0.70 | 0.70 | 1 |
| Spleen | 0.77 | 0.77 | 0.77 |
| V_ss_ (L/kg) | 0.5 | 0.5 | 0.64 |
| **Metabolism** |  |  |  |
| CL_total_ (L/h) | 25^c^ | --- | 10^c^ |
| CL_hep_ (L/h) | 22.24^d^ | --- | 7.24^d^ |
| V_max_ (pmol/min/mg protein) | 320^d^ | 194.2^e^ | 55.9^d^ |
| K_m_ (µM) | 128^f^ | 133.9^e^ | 139.8^f^ |
| CL_other_ (L/h) | 4.0^g^ | 4.0^g^ | 4.0^g^ |
| **Excretion** |  |  |  |
| CL_r_ (L/h) | 2.76^h^ | --- | 2.76^h^ |

B/P, blood-to-plasm ratio; CL_amidase_, amidase clearance; CL_hep_, hepatic clearance; CL_int,NAT2_, intrinsic clearance; CL_r_, renal clearance; CL_total_, total systemic clearance; F_a_, fraction absorbed; f_u,gut_, unbound fraction of drugs within the enterocytes, f_u,p_, fraction unbound in plasma; K_a_, absorption rate constant; K_p_, tissue-to-plasma partition coefficient, P_eff,,man_, predicted effective permeability in man; Q_Gut_, villous blood flow and permeability through the enterocyte membrane; V_ss_, volume of distribution at steady-state; FA, fast acetylator; IA, intermediate acetylator; SA, slow acetylator

^a^Gaohua et al., 2015

^b^Manually optimized for the large organs to recover the V_ss_ and concentration-time profile after i.v. administration in SA.

^c^Kinzig-Schippers et al., 2005

^d^Retrograde modeling of clinical clearance data as described in Methods section.

^e^V_max_ and K_m_ were computed from 50% of CL_int_ and K_m_ FA (*4) and SA (*5)

^f^Fukunaga et al., 2021

^g^Estimated as described in Methods section.

^h^Weber and Hein, 1979

**Table S3: Model-predicted clearance of isoniazid following administration of 300 mg single oral dose during pregnancy and compared with observed clearance from a clinical study.**

|  | **Observed clearance (L/h) ^$^** | | | **Predicted clearance (L/h)** | | |  |
| --- | --- | --- | --- | --- | --- | --- | --- |
|  |  | | | **Without enzyme activity changes (base model)** | | |  |
|  | **FA** | **IA** | **SA** |  | **FA** | **IA** | **SA** |
| PP | 66.6  (53.57 – 100.3) | 36.9  (28.25 – 58.14) | 12.0  (8.86 – 14.8) | NP | 33.9 ± 13.6 | 23.34 ± 7.83 | 12.2 ± 3.2 |
| 2T & 3T | 78.9  (62.24 – 100.3)  [16] | 47.24  (35.34 – 75.37)  [21] | 17.0  (11.8 – 27.7)  [30] | P28GW | 35.7 ± 13.1  [5] | 25.21 ± 7.73  [3] | 13.7 ± 3.4  [11] |
|  |  |  |  | **With enzyme activity changes (final model)** | | |  |
| PP | NA | NA | NA | NP | 33.9 ± 13.6 | 23.34 ± 7.83 | 12.2 ± 3.2 |
| 2T & 3T | NA | NA | NA | P28GW | 40.2 ± 13.2  [16] | 29.30 ± 8.58  [20] | 17.6 ± 4.4  [30] |

^$^Gausi *et al.,* 2022

CL, clearance; NP, non-pregnant; PP, postpartum; P28GW, pregnancy gestation week 28; NA, not applicable

Predicted CL values are presented as means ± standard deviation.

Observed CL values are presented as median (interquartile range).

The percent enzyme activity change is shown in the square brackets. For the observed CL, the clearance was compared between pregnancy (second and third trimester) and PP; for the predicted CL, the percent change represents CL change between healthy non pregnant and pregnant (28 GW) women.

**References**

Bing C, Xiaomeia C, Jinhenga L. Gene dose effect of NAT2 variants on the pharmacokinetics of isoniazid and acetylisoniazid in healthy Chinese subjects. *Drug Metabolism and Drug Interactions*. 2011;26(3).

Boxenbaum HG, Riegelman S. Determination of Isoniazid and Metabolites in Biological Fluids. *Journal of Pharmaceutical Sciences*. 1974;63(8):1191-1197

Fukunaga K, Kato K, Okusaka T, et al. Functional Characterization of the Effects of N-acetyltransferase 2 Alleles on N-acetylation of Eight Drugs and Worldwide Distribution of Substrate-Specific Diversity. *Front Genet*. 2021;12:652704.

Gaohua L, Wedagedera J, Small B, et al. Development of a Multicompartment Permeability-Limited Lung PBPK Model and Its Application in Predicting Pulmonary Pharmacokinetics of Antituberculosis Drugs: Lung PBPK Model. *CPT Pharmacometrics Syst Pharmacol*. 2015;4(10):605-613.

Gausi K, Wiesner L, Norman J, et al. Pharmacokinetics and Drug-Drug Interactions of Isoniazid and Efavirenz in Pregnant Women Living With HIV in High TB Incidence Settings: Importance of Genotyping. *Clin Pharmacol Ther*. 2021;109(4):1034-1044.

Kinzig-Schippers M, Tomalik-Scharte D, Jetter A, et al. Should We Use *N* -Acetyltransferase Type 2 Genotyping To Personalize Isoniazid Doses? *Antimicrob Agents Chemother*. 2005;49(5):1733-1738.

Kubota R, Ohno M, Hasunuma T, Iijima H, Azuma J. Dose-escalation study of isoniazid in healthy volunteers with the rapid acetylator genotype of arylamine N-acetyltransferase 2. *Eur J Clin Pharmacol*. 2007;63(10):927-933.

Yoo H, Chun Ji S, Cho JY, et al. A pilot study to investigate the utility of NAT2 genotype-guided isoniazid monotherapy regimens in NAT2 slow acetylators. *Pharmacogenetics and Genomics*. 2021;31(3):68-73.

Weber WW, Hein DW. Clinical Pharmacokinetics of Isoniazid: *Clinical Pharmacokinetics*. 1979;4(6):401-422.
